# 3D Printing of Wood Composites: State of the Art and Opportunities

**DOI:** 10.3390/polym16192827

**Published:** 2024-10-06

**Authors:** Johan Ramaux, Isabelle Ziegler-Devin, Arnaud Besserer, Cécile Nouvel

**Affiliations:** 1 Université de Lorraine, CNRS, LRGP, 54000 Nancy, France; 2 LERMAB, Université de Lorraine, INRAE, GP4W, 54000 Nancy, France; isabelle.ziegler@univ-lorraine.fr (I.Z.-D.); arnaud.besserer@univ-lorraine.fr (A.B.)

**Keywords:** 3D printing, wood fibers, wood derivatives, additive manufacturing

## Abstract

With the production of wood waste constantly on the increase, questions relating to its recycling and reuse are becoming unavoidable. The reuse of wood and its derivatives can be achieved through the production of composite materials, using wood as a reinforcement or even as the main matrix of the material. Additive manufacturing (also known as 3D printing) is an emerging and very promising process, particularly with the use of bio-based and renewable materials such as wood or its industrial derivatives. The aim of this paper is to present an overview of additive manufacturing processes using wood as a raw material and including industrial solutions. After presenting wood and its waste products, all the additive manufacturing processes using wood or its industrial derivatives will be presented. Finally, for each 3D printing process, this review will consider the current state of research, the industrial solutions that may exist, as well as the main challenges and issues that still need to be overcome.

## 1. Introduction

Wood is a natural and biological product used in many industries. Wood-based products are efficient and offer good mechanical and thermal performances. Both wood and wood-based products offer interesting mechanical performances. Wood species vary in their properties (such as density, tensile strength, or elastic modulus). For example, pine (*Pinus* sp.) has a density of around 350 kg/m³, while beech (*Fagus sylvatica*) has a density close to 560 kg/m³. Similarly, tensile strength varies from 12 MPa (pine) to 86.2 MPa (beech), and Young’s modulus from 0.1 GPa (pine) to 9.5 GPa (beech) [1,2]. Nevertheless, whatever the species, wood is suitable for processes using moderate temperatures (i.e., around 200 °C) because of its resistance to thermal degradation.

To maximize the positive carbon footprint of wood and minimize the use of fresh wood, recycling and reusing wood are interesting ways of adding value. However, in Europe, only 30% of the post-consumer wood is recycled or reused, and much of it is directly considered as waste [3] due to several factors, including heterogeneity and pollution of wood waste (adhesives, paint, presence of plastics or glues…) and the lack of recovery means. There is no international classification for wood waste (ISO 17300-1 [4] had be withdrawn in March 2021). At the European level, there is a lack of uniform regulations on waste wood among countries nowadays [5]. However, the concept of waste, and more particularly wood waste, is standardized and precisely defined according to various standards and directives. The draft catalogue of wood waste classification [6], in line with the European Waste Classification [7], contains guidelines for waste classification. In its 2008 directive [8], the European Union defines waste as “any substance or object which the holder discards or intends or is required to discard”. For wood waste, the NF EN ISO 17225-1 standard [9] provides a precise and complete classification, taking into account origin, nature, and hazardousness. Finally, the national classification systems in force in most of the European countries distinguish four wood waste classes (A to D, Table 1). These national regulations and systems systematically include two extreme classes: 1—pure biomass and 4—hazardous waste containing organochlorines or metal trace elements. Between these two extremes are two intermediate classes, which vary slightly between countries.

In 2020, the European Union and USA produced about 50 million and 60 million tons of wood waste, respectively [10,11]. To reduce waste production and prevent over-consumption of resources, finding new available paths of recycling is one of the biggest challenges of the 21st century.

Wood combustion remains a widely-used source of wood waste valorization [12,13], even if a cascading reuse would be much more favorable [3]. Particleboard remains the main way of recycling end-of-life wood [5,14,15]. However, numerous alternatives are mentioned in the literature [16] for achieving maximum recycling of wood waste, such as chemical extraction of molecules of interest and use as mulch and litter. Use as energy is also common. However, the solutions and resulting applications are closely linked to the nature of the initial wood biomass and its degree of contamination (furniture wood, which is highly contaminated, vs. sawmill waste, which is less contaminated). Chemical valorization can be considered, since different chemical components can be extracted from wood byproducts, with variable methods and solvents [17], to produce antioxidants, pharmaceutical molecules [18], and other molecules. Finally, mulch and animal bedding fabrication could be a low-tech and environmentally friendly way of reusing non-hazardous wood waste. The manufacture of wood-based composites, meaning the blending of wood in a matrix, is a virtuous and inexpensive way of recovering waste [19]. Besides, the manufacture of wood-based composites is an opportunity to recycle commonly used polymers (such as polyethylene, for example) originating from construction and demolition waste (CDW) in wood composites [20]. These composites can then be used in various segments like automotive, construction, etc. [21]. Many processes can be used to manufacture them, and among them, additive manufacturing (AM), also known as 3D printing, is emerging and attracting increasing interest due to its many advantages: low waste, objects with highly complex geometries that can be manufactured in one go and at lower cost than conventional processes, customizability, proximity to the user… Today’s target markets for 3D printing with wood composites remain niche markets. There are very few examples in the literature of large-scale use of 3D printing with wood composites.

**Figure 1:**
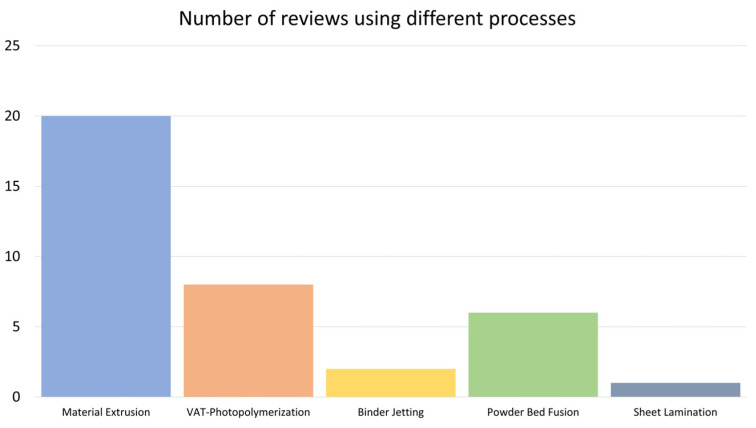
Number of reviews covering the various 3D printing processes from 1 January 2021 to 1 September 2024.

Over the last five years, a number of studies have been published on the use of wood and its derivatives in additive manufacturing (Figure 1) [1,22,23,24,25,26,27,28,29,30,31,32,33,34,35,36,37,38,39,40,41,42,43,44,45,46,47]. However, they are only focused on specific AM technologies. Besides, the state of industrialization of 3D printing technologies using wood is not mentioned in the literature. In this review, challenges and opportunities for each existing AM technology will be discussed. Industrial interest and perspectives of these process will be described as well. The diversity of wood origin and availability, as well as its physicochemical properties and its interaction with polymer matrix, will be addressed. Finally, the possibility of using wood residues in 3D printing technologies will be discussed.

## 2. Methodology of Research

**Figure 2:**
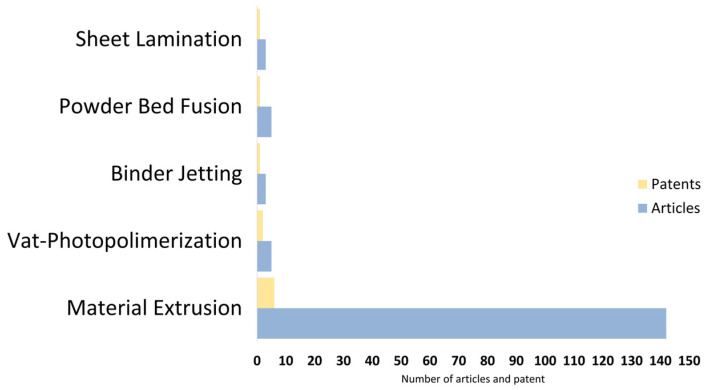
Classification of articles and patents concerning 3D printing using wood composite in the process from 1 January 2021 to 2024.

The “Web of Science” database (Clarivate) was used to process the scientific literature. Search results were listed from 1 January 2011 to 1 July 2023. The keywords “3D printing” and “additive manufacturing” were used to isolate searches dealing with 3D printing. This resulted in a total of around 73,000 papers. To focus on wood-based 3D printing, the keywords “wood” and “sawdust” were used. A total of 409 papers dealt with the subject, including 31 reviews and 318 articles. Due to the large number of articles involved, the search was refocused to between 1 January 2021 and 1 July 2023. This left 213 papers, including 174 articles. Document classification of these articles is presented in Figure 2 below. Material extrusion processes are very predominant in 3D printing research. More specifically, the Fused Filament Fabrication (FFF) process is the most studied. About 40 articles were not relevant to this review, meaning they were not about creating a wood-based composite for 3D printing. As a result, only articles published from January 2021 onwards were retained in the corpus. Other articles published before this date were also included, as they were considered significant or important. Some relevant articles published up to 2024 were also added, to include the most recent research articles.

A similar analysis of patents, using the Derwent Innovation Index (Web of Science’s patent search engine), isolated a total of 197 patents worldwide. No date restrictions were applied, as the oldest patents listed were published in 2014. For linguistic reasons, only English-language patents were processed (40 patents). 6 patents concerned innovations using the FFF process, which is the most widely used. There were also 2 patents using VAT photopolymerization and 3 other patents for other processes (Figure 2). The remaining 29 patents were not related to innovation in wood-based 3D printing.

## 3. Wood as a Resource for Additive Manufacturing

### 3.1. Structure and Composition of Wood

#### 3.1.1. Wood Chemical Structure

**Figure 3:**
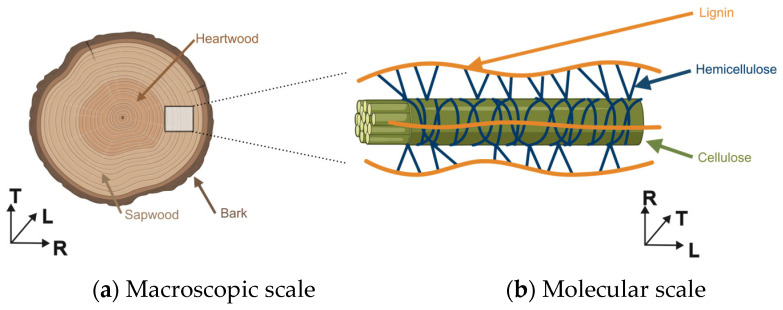
Wood chemical structure: (**a**) Macroscopic scale, (**b**) Molecular scale (R is for radial direction, L for longitudinal direction, and T for tangential direction).

Wood is a natural and biodegradable multi-scale polymer-based material. At the log level, wood can be subdivided into bark, sapwood, and heartwood (Figure 3a), which differ strongly in their cellular ultrastructure and relative content in the various bio-based macromolecules and small molecules [48]. Wood material is mainly made of lignocellulosic cell wall. The wood cell wall is essentially made up of three biopolymers: cellulose (30–50 wt.%), lignin (25–40 wt.%), and hemicellulose (20–30 wt.%). These three biopolymers intermingle to form a complex, highly resistant, three-dimensional structure, held together by both hydrogen and covalent bonds (Figure 3b). The components are present in a different ratio [1,35,49] in both softwood and hardwood but also between individuals of the same species. In a very poor quantity (<5 wt.%), temperate wood contains other molecules, known as extractives, which can be extracted with various solvents (water, alcohol, toluene, etc.). Those extractives can be aromatic phenolic compounds, aliphatic compounds (fats and waxes), and terpenes and terpenoids and are high value-added molecules for specific segments such as cosmetics, pharmaceuticals, or the agri-food sector [17].

##### Cellulose

**Figure 4:**
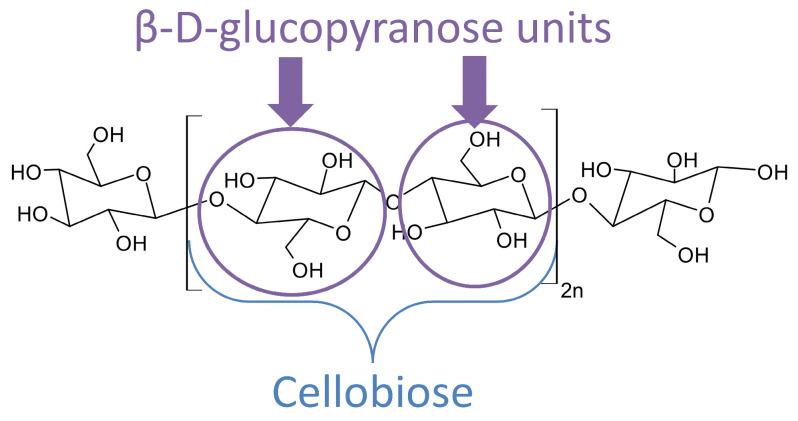
Chemical structure of cellulose, with n the polymerization degree and cellobiose the repetitive unit.

Cellulose (Figure 4) is the most important macromolecule in plant cell walls (35–50 wt.%) [49], making it the most abundant biopolymer on Earth [50]. Cellulose is a semi-crystalline polysaccharide whose repeating unit is cellobiose. Cellobiose consists of two monomer units of β-D-glucopyranose [51]. This polymer is composed of a long polysaccharide chain with a high degree of polymerization (about 10,000 glucose units in wood), inducing a high molecular weight. Cellulose is a semi-crystalline polymer with crystalline and amorphous zones. Various methods are used to measure crystallinity [52]. This crystallinity has an impact on the mechanical strength of the plant and its individual cells [53]. Due to the presence of three OH groups per glucoside unit, intracatenary (between cellulose units) and intercatenary (between cellulose chains) hydrogen bonds are formed, leading to microfibrils composed of 30 to 40 cellulose chains, which aggregate to form fibrils and then rigid fibers. All these interactions form complex and organized networks, giving the cellulose polymer high density, stability, and rigidity. Cellulose and its derivatives are very common material for various industrial sectors like the paper industry, textiles, insulation… [54].

##### Hemicellulose

**Figure 5:**
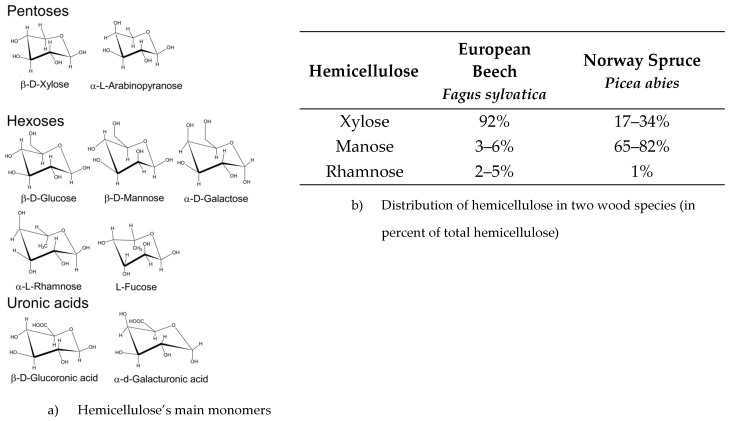
Main monomers of hemicellulose (**a**) and an example of hemicellulose distribution in two different wood species according Marynowski et al [55] (**b**).

Hemicellulose is a very abundant polysaccharide in plants, with about 20–30 wt.% [49]. Hemicelluloses are amorphous, heterogeneous, branched polymers of relatively low molecular weight, with a polymerization degree between 50 and 200. Unlike cellulose, they are easily hydrolyzed and contain various monomer units (Figure 5) consisting of five-carbon monosaccharides (xylose and arabinose) as well as six-carbon monosaccharides (glucose, mannose, galactose, fucose) and uronic acids (galacturonic and glucuronic acids). The composition of hemicellulose depends on the wood species [55]. The combination of cellulose and hemicellulose is called holocellulose. The most important role of hemicellulose is its contribution to cell wall reinforcement through interaction with cellulose, and, in some walls, with lignin. The ratio between mannose and xylose is significatively different for softwood and hardwood [55].

##### Lignin

**Figure 6:**
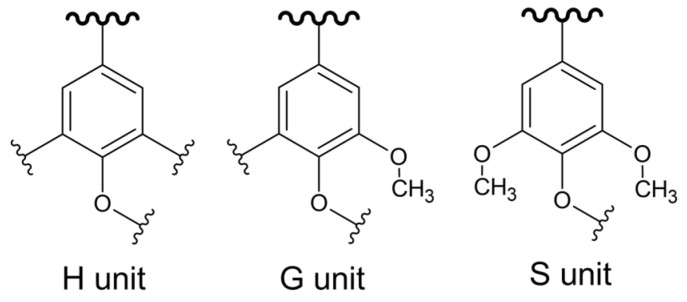
Lignin monomer units.

Lignin is the first renewable aromatic polymer on Earth [56]. The term lignin covers, in fact, a large group of biopolymers with aromatic skeletons. These polymers are mainly present in cell walls, making them impermeable and rigid. This prevents the sap, composed mainly of water and nutrients, from passing through the cell wall, while providing the rigidity needed to hold the plant in position. Lignin is composed of three phenylpropanoid units (Figure 6), syringyl (S), guaiacyl (G), and p-hydroxyphenyl (H), which are linked mainly through ether or carbon–carbon bonds such as β–O–4, β–β, etc.

Lignin is a recalcitrant polymer embedding cell wall polysaccharides. This reduces the carbohydrates’ accessibility during microbial degradation. However, numerous bacteria and filamentous fungi have developed biochemical routes to use lignin as a carbon source for their primary metabolism [57,58,59]. Lignin is an amorphous polymer that, like cellulose, does not take on a particular predefined form. It occupies the space left free by the other constituents of the plant wall, giving the lignified wall its rigidity and impermeability. In paper industries, lignin is currently considered a waste, and its main use is for energy production.

##### Wood Sources for 3D Printing

Growing interest in the use of wood in biobased material production comes from the abundance and the large range of possible wood-derived products. Indeed, wood can be used to produce resin and synthons by biorefinery-based processes [60,61]. The forest-timber sector is indeed based on a circular economy model. The wood processing from the log to the timber or numerous wood derived products (wood panel, synthon molecules, resin, and binder or wood-plastic composites) generates side- and byproducts (sawdust, bark, off-cuts…) that are currently considered waste and mostly burnt or landfilled. Post-consumer wood products are in a similar situation. Taken together, according to the wood residues data from the Food and Agriculture Organization (FAO), wood waste is a huge stream for a new raw material, with more than 240 million m³ in the world in 2020 [62]. Thus, there is high potential to create added value on wood waste by cascade valorization [3,63]. However, the physicochemical properties, the availability, and the price of the wood materials that could be used for new products are very diverse and may require sorting to be processed properly. Besides the differences linked to the wood compartments and the physical nature of the wood material (solid, particle, fiber…), the presence of additives not naturally present in wood must be considered as well for both energy and material recovery.

The simplest wood wastes to recycle are machining off-cuts, sawdust, shavings, and other wastes containing no substance other than wood. These wastes, known as A class, can be used in the particleboard industry or for the extraction of high value-added molecules. Wood waste that contains contaminants can be treated, or reused and recycled in certain ways, but represents an increasingly important source. This wood waste, known as “B and C classes” wood, comes mainly from the furniture industry. Another stream is made of construction and demolition waste (CDW), classified as “C and D classes”. This wood waste can contain substances of very high concern (SVHC). In Europe, these SVHC are defined by the European Chemical Agency, and thresholds are defined by standards such as those of the European Panel Federation [64]. In wood, heavy metals (arsenate, copper, cadmium, chromium, lead) and organic contaminants (creosote, pentachlorophenol, halogenate compounds, formaldehyde [65,66]) are the mostly common contaminants that have to be removed to allow an eco-friendly valorization of wood waste. Currently, these wood wastes are landfilled or burnt in dedicated installations [67]. Due to social habits’ evolution and the increase of furniture turnover, medium-density fiberboard (MDF) waste amounts are increasing over years, which raises recycling issues [68,69]. Wood waste and wood derivatives can therefore become good candidates for additive manufacturing. There are many possibilities for adopting wood in additive manufacturing; this is due to the huge wood species availability and the different kinds of morphology that exist. Indeed, wood can be found with different grain sizes suitable for different 3D printing processes. Wood is well-suited to 3D printing, as the possibilities for including it in composites are numerous, which is not the case for all materials. Moreover, the various constituents of wood waste or raw wood can be extracted to offer products suitable for different needs. In different AM processes where there is a need for heat, the thermal degradation temperature of wood is sufficiently high enough to be used. Thermal degradation occurs at temperatures above 200 °C [70,71]. That is why wood-based composites can be found in five of the seven additive manufacturing process categories (Figure 1).

#### 3.1.2. A Wood Waste Example: Case of Medium Density Fiberboard (MDF)

MDF panels are widely used in the furniture industry. They are made from wood fibers that are glued and then hot-pressed. For this type of panel, urea and formaldehyde-based adhesives are used, with contents of 5–9 wt.%. The panels are then pressed at temperatures between 180 °C and 220 °C and at pressures of 50–60 bar for a few seconds (order of magnitude of 3–4 s/mm thickness) [72]. Other types of adhesives are tending to be developed, with the aim of limiting formaldehyde content [73]. These glues incorporate melamine-based compounds or biobased binders [74]. MDF panels are renowned for being machinable, paintable, and affordable. Nevertheless, their lifespan remains relatively short; indeed, as Irle et al. [68] point out, over 40 years, more than 90% of MDF panels produced ended up as waste. The presence of formaldehyde in panels makes their energy recovery at the end of their life very complicated. Indeed, during combustion, numerous molecules are emitted, such as carbon monoxide, nitrogen oxides, and sulfur oxides [75]. Removal of resin and additives from MDF wood and efficient recycling are main challenges. Because formaldehyde urea resin can be hydrolyzed, hydrothermal treatment appears to be a promising pretreatment route for various subsequent valorizations [69,76,77]. However, in the 3D printing process, fibers are hardly usable. Fine particles, such as sawdust recovered from machining, are the best candidates for use as fillers in printing composites. Taken together, the increase of MDF production, the growing interest for AM using bio-based and biodegradable polymers, and the challenge of prolonging the CO_2_ fixation time in wood as much as possible support the use of sawdust in the printing composite.

#### 3.1.3. Wood-Based Composites

A composite is made of two or more components with dissimilar chemical or physical properties that are not naturally miscible, and their properties are enhanced over those of the components taken separately. They are composed of at least a matrix and a reinforcement agent. Wood-based composite materials, such as particle board or medium density fiberboard, are available on the market and well documented in the literature [5,78]. Since the beginning of the 1970s, new kind of wood composites have appeared, made of wood and a polymer. These new composites are called Wood Plastic Composites (WPCs) [21,79]. WPCs are often obtained by injection molding or extrusion processes [80,81]. With the recent increase of interest in 3D printing, wood-based composites for 3D printing are more and more studied in literature, as in [25,27,41], for example. This reflects a major interest in this field. The polymer matrix can vary depending on the process involved. Thanks to the wide range of processes for producing wood composites for 3D printing, a wide range of markets have been targeted, such as sound insulation [82], rapid prototyping, etc. Historically, 3D printing, and particularly extrusion printing, has been used for rapid prototyping. However, new markets are increasingly opening, offering higher-value-added and more-complex products while lowering production costs for small- and medium-sized production batches such as furniture and manufacturing [83]. 3D printing thus makes it possible to use a wide range of lignocellulosic reinforcement sources and matrix types, as illustrated in Table 2. The resulting composites are therefore highly diversified and can meet the needs of a wide range of innovative markets. Finally, wood components can be extracted and incorporated in wood-based composites such as nanocrystalline cellulose, but this composite will not be discussed in this review.

### 3.2. Additive Manufacturing Generalities

#### 3.2.1. Working Principle of 3D Printing

**Figure 7:**
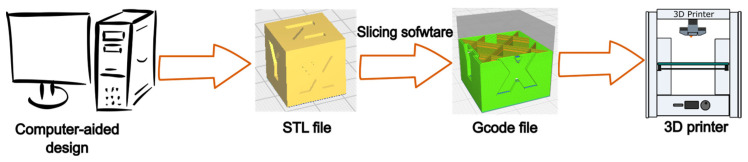
3D printing process diagram.

As defined by the ISO/ASTM 52 900 standard [95], AM, also known as 3D printing, is a “process of joining materials to make parts from 3D model data, usually layer upon layer”. It all starts with the creation of the model, using computer-aided design software to generate a file (STL, OBJ, 3DS…). This file is then loaded into the slicing software (commonly known as a “slicer”). The purpose of this software is to transform the drawn model into a succession of layers that will be printed. Finally, once the slicing has been completed, the resulting Gcode file is fed into the 3D printer, which reads each line and produces the final object. Figure 7 shows a schematic diagram of this process.

#### 3.2.2. 3D Printing Processes

**Figure 8:**
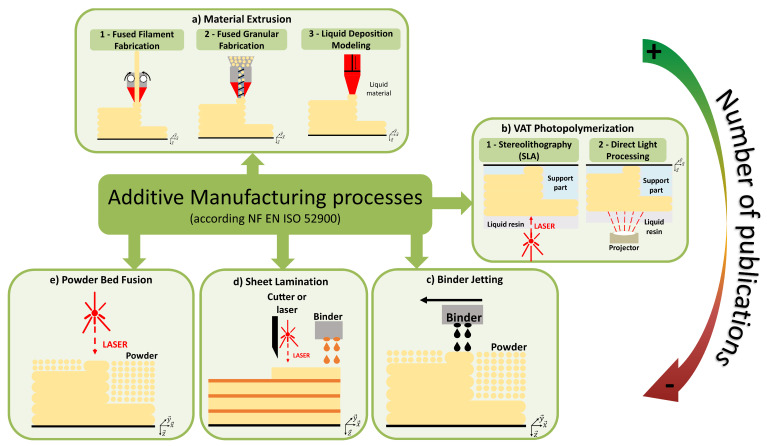
Additive manufacturing classes.

According to the ISO 52900 standard, seven different 3D printing processes can be identified, some of which are illustrated in Figure 8. The most popular 3D printing process is material extrusion. In this process, material is extruded through an orifice. Different types of material can be extruded, such as gels or viscous materials at room temperature, or thermoplastic polymers. In the case of gel, the process is called Liquid Deposition Modeling (LDM, illustrated in Figure 8(a3)), whereas in the latter case, the polymer is heated inside a printing head through a heating element and then extruded through a nozzle. Two versions of this process are available. The polymer is printed from a filament, in the case of Fused Filament Fabrication (FFF, illustrated in Figure 8(a1)), also know under the patented name of “Fused Deposition Modelling” (FDM), whereas for Fused Granular Fabrication (FGF, illustrated in Figure 8(a2)), the material is in pellet form.

The oldest 3D printing process (VAT photopolymerization) was discovered simultaneously by Jean Claude André’s [96] and Charles W. Hull’s [97] research teams in 1986. It consists of the polymerization of a UV resin. It involves selective layer-by-layer curing of a liquid resin contained in a vat through targeted photo-polymerization using an energy source (laser, screen, or LED). Two processes predominate: the first uses a laser source to photopolymerize the resin (Stereolithography (SLA), illustrated in Figure 8(b1)), while the second uses a screen (Digital Light Processing (DLP), illustrated in Figure 8(b2)). Once the process is completed, a post-polymerization is necessary to consolidate the final object.

Other processes use a powder material as the starting material, like binder jetting, first described by Sachs and al. [98] (Figure 8c). For each layer of powder, the binder is sprayed where required. Extra powder is collected and reused for future applications. Post-treatment is necessary to remove unbound powder. In addition, annealing is often carried out to improve the performance of the resulting parts. Other post-treatments can be used, including debinding, sintering, or infiltration [99]. Even if binder jetting is a mature and industrialized process, the mechanical properties of the parts obtained are still generally weak [100].

Sheet lamination (Figure 8d) operates in two stages. The first is to create the various layers by conventional manufacturing methods (machining, laser cutting…) or by additive manufacturing. Once the layers have been obtained, they must be bonded together with a binder. This process allows the use of a wide range of materials (for example wood panels or sheets) and can open a variety of markets (furniture, custom packaging, etc.).

The powder bed fusion (Figure 8e) process is close to binder jetting (Figure 8c). The powder is spread out on the printer plate, but instead of spraying a binder where needed, a laser fuses the powder point by point on each layer. The resulting parts have a granular appearance, typical of the sintering process. The remaining powder can also be recycled, but to a lesser extent, because of its partial degradation during the process. Like binder jetting, post-treatment is necessary. The name of the technology is defined by the type of fusion source used. Fusion with a laser [101] is called Selective Laser Sintering (SLS), used for polymers.

## 4. Use of Wood in Additive Manufacturing

For each of the printing processes described above, the state of the art in current research and potential applications will be discussed, including the challenges to specifically printing wood or wood-based material. The processes with the fewest publications will be discussed first.

### 4.1. Binder Jetting

#### 4.1.1. Added Value of Binder Jetting

The most famous example of binder jetting with wood powder is the technology developed by Forust (owned by Desktop Metal). Their binder jetting 3D printing process is aimed at various high-value-added product markets, such as luxury car interiors, consumer goods, architecture, and furniture [102,103]. This printer [104] can realize a 200 µm layer of 350 × 220 mm in an hour, but products may feel grainy to the touch. Final parts made with binder jetting can have a higher added value, which can be a way of improvement for new applications.

#### 4.1.2. Mechanical and End-Use Properties

The literature about using binder jetting with wood powder is extremely poor (only four valuable results from 2011 to 2023 on Web of Science). One review focuses on binder jetting in the construction industry [105]. Another study investigates the use of wood particles produced by house borers or termites, with promising results. The parts obtained only demonstrated the feasibility of the process, as their mechanical strength was not good enough for the authors [84]. Finally, an investigation about fiber surface and binder requirements has been carried out by Evdokimov et al. [106]. This research seems to show that certain wood particles, and consequently certain species, are more easily suitable than others for the binder jetting process because of their greater specific surface area.

Lastly, binder jetting has been considered coupled with sheet lamination process. In this new process, named Individual Layer Fabrication (ILF) or Individual Layer Modelling (ILM), wood chips or sawdust are sprayed in a thin layer, binder is sprayed onto required places, and then mechanical pressure is applied (with or without heat). Polyvinyl acetate and urea formaldehyde adhesives were tested as binders with wood flour [107], but no data are presented about Volatile Organic Compound (VOC) emissions. This process holds great promise for reusing wood chips instead of powder, even if, for the moment, it is only a proof of concept. Thus, Henke [108] has used 0.8 to 1.1 mm wood chips and a synthetic adhesive based on polyvinyl acetate.

Forust’s solution seems to be the only turnkey solution. A patent of Z Corporation [109] (now owned by 3D Systems) claims a solution of binder jetting technology suitable for wood powder and many other powders (metal, ceramic, etc.).

#### 4.1.3. Challenges for Future Development of Binder Jetting

The challenges for binder jetting using wood powder are numerous. Biobased binders can be a very important step in binder jetting development. This should make it possible to reduce the carbon footprint of this process and offer new alternatives. The use of mixed wood species is an issue that must be fixed too.

### 4.2. Sheet Lamination

As previously explained, sheet lamination is realized in two steps. The first one is the deposition of layers. This can be done with subtractive or additive manufacturing. Once layers are produced, the second and final step is to assemble the layers together.

#### Potential Markets

This process makes it possible to use very low-cost materials to produce parts with higher added value. The most widely used (from lignocellulosic materials) are paper, cardboard, and wood panels. Although not very common in industry, there are many possible applications for them. Products made by Stratoconception (as described in patents of CIRTES, France) [110,111] illustrate this, enabling the production of custom packaging (cardboard), furniture elements, or prototypes. 3D printing using the layer lamination process can also meet the growing challenges of large-scale printing using wood [112]. To obtain the final parts, the sheet laminating printing process requires a material that can be cut. The materials used are numerous, so there are many possibilities for obtaining wood-based products. Although sheets are traditionally cut by a cutter or laser, it is also possible to produce them using 3D printing, such as binder jetting [108,113]. However, whatever the sheet types, the final properties are highly dependent on the amount of binder between the different layers. Maximum tensile strength (about 34 MPa) has been achieved with 17 wt.% binder [113].

### 4.3. Powder Bed Fusion

For each layer, a little bit of powder (e.g., wood) is spread out on the bottom layer. A heat source fuses the material point by point according to the 3D file.

#### 4.3.1. Added Value of Powder Bed Fusion

Using wood as a reinforcement for polymer-based powder, SLS finds applications for high-value-added technical parts. High-porosity carbon electrodes were successfully printed [93] from pine powder and phenolic resin. After printing, the parts were pyrolyzed under nitrogen to obtain the carbon electrodes. Porous wood composites for wave absorption have also been printed with the SLS process, using phenolic resin and cedar wood [114].

#### 4.3.2. Properties and Recycling Issues

The SLS printing process can be used to print a wide range of parts. However, for mass-produced parts, this technology does not deliver mechanical performance comparable to 3D printing processes such as FFF [92]. To improve the mechanical strength, it is possible to vary the parameters of the 3D printer to enable better cohesion between the matrix and the reinforcement. The laser intensity used in the process has a significant impact on tensile and flexural strength values [115]. Indeed, a luminous flux intensity (irradiance) greater than 311 W/mm² has a negative effect, showing that there is an optimum to be determined. Another solution for improving mechanical performance is to add a compatibilizer, whose role is to bind the polymer matrix to the lignocellulosic reinforcement. With the powder bed fusion process, the addition of carbon nanotubes [92] in very low proportions (0.1 wt.%) enables tensile and flexural strengths to be increased significantly (+176% and +328%, respectively). The use of wood in powder bed fusion is quite widespread. For instance, the use of 20 wt.% of pine powder shows very interesting results, according to the authors [91]. A loading rate of up to 50 wt.% of pine powder has been tested for the manufacture of carbon electrodes [93]. A study on the use of lignin shows that it can be used up to 40 wt.% [116].

### 4.4. Vat Photopolymerization

Vat photopolymerization involves using a tank (vat) filled with liquid resin of relatively low viscosity (less than 1000 mPa.s) [117,118]. The resin is then polymerized layer by layer according to the desired 3D model. Different processes are used, differing in the means employed to initiate polymerization of the resin. The oldest photopolymerization process uses a laser (stereolithography—SLA) to polymerize the vat layer by layer [96,97]. Other processes have emerged, notably using a screen (Digital Light Processing—DLP) to polymerize the entire layer at once. It is already known that vat light-curing enables the use of composites [119]. Vat photopolymerization is mainly used in the jewelry and dental sectors. However, until now, there has been no commercial use with wood or wood derivatives. Vat photopolymerization is a process that has not been much studied in the context of wood composites, discussed in only five articles, including four on the DLP process and only one on SLA.

#### 4.4.1. Interests and Potential Markets

This 3D printing process is commonly used in applications requiring high precision. Commercially available resins offer high resolutions of a few tens of microns [120]. The benefits of introducing wood particles into light-curing resins are manifold, enabling the production of high-resolution prototypes or parts, while reducing the cost of the materials used.

#### 4.4.2. Properties of the Composites Obtained

Three types of reinforcement have been tested for vat light-curing. The use of lignin is the subject of two articles, while the use of wood powder concerns three articles.

Poplar wood was used to increase tensile strength by 17% to 24.7 MPa with just 1 wt.% load [121]. By adding 2 wt.% poplar, a maximum increase in Young’s modulus (+90% compared with controls) but a decrease in tensile strength (compared with incorporating 1% wood) was observed. Higher loading rates of up to 10% were successfully achieved using incense cedar [122] as well as other woods [123]. Microwave pre-treatment of the wood powder helped reduce water absorption.

The incorporation of lignin in a UV-curable resin has also been studied in different ways. At a very low content (0.2–1 wt%), kraft lignin (from the paper industry) was incorporated in Formlabs Clear Resin [124,125]. Tensile strength and Young’s modulus increased by 52% and 26%, respectively [94], when using the post-cured process. Some researchers prepared the resin themselves and used a silver birch lignin load rate a little bit higher (4 wt.%). The technology they used was DLP technology [126]. As a result, they found that the incorporation of a maximum of 3 wt% resulted in reproducible prints.

#### 4.4.3. Challenges for Future Development of VAT-Photopolymerization

Only untreated wood and lignin were studied for photopolymerization in a tank. The wood loading rate is much lower than with other technologies such as FDM or BJ. Thus the maximum amount of poplar wood tested was 10 wt% [121] and only 4 wt% for the lignin incorporation [126]. The limitation of lignin content is supposed to come from the UV strong absorption of lignin at the wavelength of an SLA laser (405 nm).

Methacrylate-based resins used in the DLP process are hydrophobic [123], making them very difficult to be melted with the hydrophilic wood. So, one of the main challenges for future development is to improve compatibility between both matrices and wood residues. Besides, methacrylate resins are petro-sourced products with a big impact on the environment. This is why obtaining bio-sourced resins is a promising avenue for future research. Even if there are many manufacturers producing 3D printers for VAT photopolymerization or UV-curable resin, no data have been found to ascertain that wood-based curable resin is commercially available. Resin viscosity increases by a factor of seven with the addition of just 4% lignin [126]. Therefore, viscosity could be a problem at higher loading rates. As lignin seems to be a problem for curing, wood delignification seems to have good results with the incorporation of 10 wt.% delignated wood flour [127]

### 4.5. Material Extrusion

Material extrusion 3D printing involves extruding a material through an orifice called a nozzle. There are two main types of material extrusion: room-temperature extrusion and high-temperature extrusion (over 100 °C). Room-temperature printable materials consist of gels filled with wood particles or derivatives (most often cellulose). This type of extrusion is also used in the construction industry for concrete-based composites [128]. The term Liquid Deposition Modeling (LDM) is most often used to name such a process. Hot extrusion involves melting a material above its melting temperature. The materials used are thermoplastic polymers. There are two types of process on the market. Fused Filament Fabrication (FFF), also known as Fused Deposition Modeling (FDM), uses a filament to feed the printing head. The second is Fused Granular Fabrication (FGF), which uses thermoplastic pellets rather than a filament inside the printing head. Material extrusion is a low-cost 3D printing process that can be used in many sectors. Furthermore, since it is easy to implement, it is the most popular with the public. According to Vaisanen et al., FFF 3D printing using wood composite seems no more dangerous than with virgin PLA [129]. This process has also been modeled to optimize its operating parameters [130,131,132].

#### 4.5.1. Added Value and Industrial Markets

Although this process has historically been used exclusively for rapid prototyping, many other sectors are now also taking advantage of 3D extrusion printing processes. In recent years, several case studies have been reported in the literature. 3D printing makes it possible to optimize materials and thus create lighter, customized sandwich panels using wood composites for a variety of applications [133,134,135,136,137,138]. As wood is well known for its insulating properties, investigations have been carried out into the creation of wood-based acoustic panels, by Sekar et al. [82,139,140,141,142], or as electrical insulation [143]. If the hygroscopic character of wood can be a disadvantage for wood-composites, it has been leveraged using 3D printing to create hygroscopic actuators [144] and custom-designed natural desiccants [145]. Various demonstration items have also been printed [146,147], such as spoons [88] or composite crates [148]. Composites with advanced properties, such as antimicrobial [149] and phosphorescent [150,151] properties, or for the development of specific reactors [152], have also been developed using wood-based composites. Shape memory materials have also been developed [153,154,155], paving the way for 4D. The materials developed as part of the 4D project use the hygroscopic nature of wood to change shape in a controlled way after printing [156]. This is made possible using LDM and a cellulose hydrogel. Large-scale printing is also currently being developed using five-axis robotized brats [157] or, in the construction field, using an LDM process. In the latter case, the composite consists of concrete reinforced with wood fibers [158]. Finally, the reuse of 3D printing waste to create new 3D prints with these composites is only just beginning to be studied [159].

#### 4.5.2. Room Temperature Extrusion—LDM

The LDM process is based on the curing of thermosetting polymers at room temperature. This process is the only one to use thermosetting polymers with material extrusion. Research into LDM uses hydrogels based on cellulose [160,161,162,163], urea-formaldehyde resins [164], or epoxy resins [165], the last enabling the use of up to 71% wood, which is more than in case of FFF thermoplastics. The use of cellulose, and more specifically nanocellulose, enables flexural strengths in excess of 80 MPa to be achieved [166]. Finally, it has been shown that hydrogels containing hemicellulose or lignin are also possible, while implementing this system on a five-axis robot [167].

#### 4.5.3. Hot Extrusion—FFF/FDM and FGF

3D printing processes using thermoplastic polymers are widely studied and used. This is mainly due to the affordable cost of the technology, which can be found among the public. Numerous polymers are used, but PLA remains the most widely studied and most widely used. This is mainly due to the printability of this material, thanks to its moderate melting temperature (170–180 °C), low shrinkage during cooling, and low toxicity [168], which makes the residues and dust generated during printing less of a problem [169]. Another main advantage of PLA is that it is bio-sourced and usually produced from corn or sugar cane. That is why the main articles deal with PLA.

##### Commonly Used Thermoplastic Polymers

As mentioned, PLA is the most widely used polymer and, consequently, the most studied in the literature. Other polymers studied include ABS, polyethylene (PE), and polypropylene (PP). Despite its many advantages, PLA has several drawbacks. As a polyester, its sensitivity to water can cause the material to age and deteriorate. ABS is a blend of copolymers based on a poly(styrene-co-acrylonitrile) copolymer in which polybutadiene nodules are dispersed, whose formulation depends on the desired properties of use of the final product [170]. ABS’s main advantage over PLA is its resistance to water and aging. Nevertheless, as well as being a petro-based polymer, it releases several harmful compounds during the printing process [171,172], so special precautions, such as machine fairings, must be taken. That is why there are only a few relevant publications regarding ABS polymers and wood or wood derivatives. PE is a widely used petroleum-based polyester. Its melting temperature, around 240 °C, makes it highly resistant to heat. However, belonging to the same family (polyester) as PLA, it is also sensitive to hydrolysis. A variant of polyethylene, poly(ethylene terephthalate), commonly used in plastic bottles, has also been studied, with a view to promoting its recycling and adding value to this waste. Finally, other polymers like poly(butylene succinate) [173] or polypropylene (PP) can be used. PP is another petro-sourced polymer [174] belonging to the same family as PE. However, its melting temperature is lower (150–160 °C).

##### Mechanical Properties Obtained

Tensile strength is the property most often measured, and thus reported, in the literature. For example, Kariz [175] found that tensile strength increases until 10% of the loading rate (55 MPa to 57 MPa) but decreases for higher loading rates (30 MPa with 50% wood). Using a similar loading rate, Yu [176], with 11% of Astragalus byproduct, obtained between 17.36 and 23.51 MPa, depending on printing parameters. Using a 30% wood filament, Ayrilmis showed that layer thickness has an impact on tensile strength (increasing layer thickness, decrease tensile strength) [177]. Narlıoğlu reported very poor value compared to other authors (around 8 MPa for a composite with 20% of black pine). However, many authors found that the tensile strength of composite was lower that for virgin PLA. This can be due to poor interfacial bonding between components. Different fibers and wood species have been tested, with differing results. Several studies used a commercial filament as the base material: ColorFabb’s WoodFill, containing 15% wood [82,178,179], or its 30%-filled version [180]. The Hatchbox filament was also tested in one study [181]. Other studies have used hardwood [182,183,184] or softwood (fir) [185]. Wood dust at a 10% level and a recycled ABS shows interesting results [186], with a stable strength resistance (23.69 ± 1.1 MPa with 2.5% of dust and 22.71 ± 1.2 MPa with 10% dust). In conclusion, tensile strength depends on different factors such as wood species and residues, process, printing parameters, and, finally, matrix parameters. As PLA is the most widely used polymer in FFF printing, most articles deal with it. Nevertheless, there are other studies using and detailing certain mechanical properties of ABS [187], PE [188,189,190], and PP [191,192,193]. PP and HDPE are well known to present 3D printing troubles like warping, shrinkage, and adhesion [194,195,196]. However, it is a very common polymer, which can easily be found in households (detergent bottles, oil bottles…). Adding wood powder into a PP composite leads to decreased printing issues, especially shrinkage and warping [87]. PET and all polymers with high levels of crystallinity are more likely to give rise to shrinkage problems during printing [197].

The study [177] also shows a link between layer thickness and mechanical properties (tensile and modulus strength and bending). This is explained by the authors by the presence of gaps between layers, increasing water absorption and decreasing strength and bonding resistance. These results have also been found by Yu and al [176]. The behavior of the composite can be affected by printing parameters from the slicer [198,199]. Printing parameters have been studied to determine the evolution of different properties. The printing temperature (200–220 °C), filling density (60–100%), printing speed (50–70 mm/s), and layer thickness (0.1–0.2) seem to have an effect on mechanical properties (flexural and tensile strength) [176]. Increasing fiber lengths from 74 µm to 125 leads to a decrease of almost 15% of tensile strength [200]. Adding 5% of silane increases tensile strength. However, adding wood leads to a decrease of mechanical properties, as with the research of Tao et al. [86]. With longer fibers (106–425 µm), tensile strength also decreases with the addition of wood [201]. This study also demonstrates that the additive manufacturing process has better tensile strength than compression molding. Mechanical properties of composites can be very different from one paper to another. This can be explained by several parameters, such as matrix polymer use of PLA, ABS, or other thermoplastics. Printing parameters such as orientation or layer thickness lead to various results. Estakhrianhaghighi et al. demonstrate that infill pattern can lead to differences in flexural properties [202]. Finally, differences can be explained by the wide variety of wood species used. However, all results are similar on one point: above a certain loading rate (generally 20–30%), mechanical properties decrease, meaning that interactions are not perfect between wood and matrices.

##### Need for Compatibilizer

**Figure 9:**
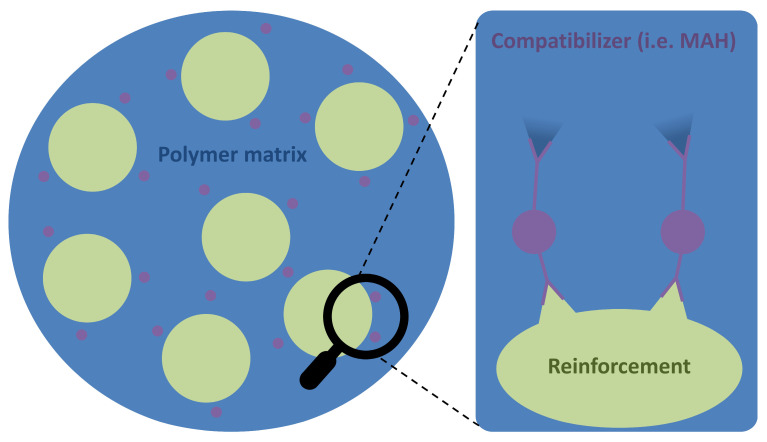
Compatibilization concept for a reactive compatibilizer.

Mixing matrices and loads can lead to poor properties. This can be due to poor interaction between polymers and wood. To reduce interaction issues, it is possible to include compatibilizers. The compatibilizers physically interact or react at the interfaces between the wood material and the incompatible polymer. The case of a reactive compatibilizer, like MAH [203], is shown on Figure 9. Different kind of compatibilizer can be used. The best one is a molecule which will create new interactions between the matrix and the load. Weak bonds (hydrogen, Van der Waals) can also be involved. Moreover, to improve efficiency, reactive groups can be grafted along the chain or at the end of the chain. Several compatibilizers have been studied, some oil-based and others bio-based. Anhydride maleic (MAH), an oil-based component, has been mixed with LDPE, improving tensile strength [204,205]. MAH and PLA have also been tested together with the incorporation of MAH in PLA, obtaining PLA-g-MAH. As a result, with 30% of PLA-g-MAH, bending and tensile strength increased [206]. PLA-g-MAH can reduce water absorption, compared to a composite without MAH [206]. The incorporation of MAH without being grafted onto PLA has also been tested, but it is less effective, leading to a drop in tensile, flexural, or impact strength [207]. TPU (also oil-based) has also been investigated in a PLA/wood composite as a compatibilizer. Results show an increase in impact strength of 51%, but also in tensile strength (34%) and flexural strength. Poplar wood has been used (10 wt%), as well as polyethylene wax (0.5 wt%) as a lubricant to improve processability [208]. As explained earlier, the purpose of a compatibilizer is to create new bonds between the matrix and load. Lignin has been studied not as a compatibilizer but as a loading, and it appears to make an interaction with PLA [209]. It could therefore be possible to develop lignin as a coupling agent. Other marginal treatments have been tested to improve the performance of 3D-printed wood composites. Heat treatment of wood involves heating it for several hours at temperatures higher than 200 °C. This treatment has been applied to composites coupled with silver nanoparticles that impart antimicrobial properties to the composite [149]. Heat treatment has also been studied, making it possible to increase the filler content of composites while maintaining tensile strength [210]. Finally, post-treatment by hot compression in the presence of salt [179] increased tensile strength but reduced elongation at the break.

##### Surface Characteristics

All the articles analyzing the surface roughness of wood-based composites seem to show that the addition of wood particles leads to an increase in the material’s roughness. The roughness of a material, and therefore its surface appearance, can be measured using a roughness meter, which provides indicators such as arithmetic roughness (Ra). This roughness corresponds to the arithmetic mean of the surface defects observed. In a process such as FFF, the roughness of the printed parts is due to the superposition of filament deposition to form the part. Overall, the addition of wood to a thermoplastic for 3D printing results in an increase in surface roughness. Surface roughness (measured by arithmetic rugosity—Ra) significantly increases when wood is added into PLA.

Depending on the printing conditions, surface roughness can vary, even on a virgin polymer. For example, some studies report an Ra of 4 µm for virgin PLA [211], while others report an Ra of 62 µm [212]. To reduce roughness and make the surface smoother, several studies suggest reducing the layer thickness during 3D printing [134,135,136,213]. Extrusion temperature, which could help to make the material smoother because it is more fluid, does not seem to have any effect [214] Yang et al. measured an Ra of only 6 µm regardless of the extrusion temperature (tested between 200 °C and 230 °C) with a composite containing 40 wt% of red cedar. However, surface topographies present gaps of more than 6 µm. A study [212] comparing PLA filament, ABS filament, and a commercial filament containing 20% bamboo (BambooFill filament) concluded that BambooFill filament has the lowest roughness (53.57 µm), followed by ABS and then PLA (61.92 µm). The authors explain this by the homogenization of the pure polymer blend, leading to a higher roughness.

##### Rheological Properties

Rheology is the study of the flow of material under stress. This is related to the behavior of the melted material in the extruder or the 3D printing. In a first analysis, the Melt Flow Index (MFI) can be very useful, because it is a rapid measure at a low shear rate. Adding wood or wood derivatives such as lignin leads to an increase of MFI and a decrease of melt viscosity. Adding lignin in PLA [215] will have two consequences: an increase of MFI from 2.2 (neat PLA) to 26.4 (30% lignin) g/10 min but also an increase in the dispersion of values (standard deviation of 0.1 g/10 min for virgin PLA v.s. 5.0 g/10 min with 30% lignin). However, using oak flour, Yatigala et al. [216] obtained opposite results with an identical loading rate (30%). The MFI of their composite was lower than that of virgin PLA. A decrease of MFI means a strong interaction between polymer and wood. Similar results were found by Petchwattana et al. [200] in their study of teak wood (5% loading rate) at different mesh sizes. For both 74 µm and 125 µm fibers, the MFI decreases. With low proportions of wood and recycled ABS, an increase in MFI can be observed up to 3% wood, then a decrease up to 5%. Recycled ABS has a much higher MFI than virgin ABS, which is due to the different compositions of the thermoplastics (different monomer percentages) [186].

Rheology can also be used to measure the viscosity of the material as a function of the shear applied. This can be useful for predicting material behavior in a 3D printer, an environment in which the composite is subjected to high shear rates. The viscosity of the composite is highly dependent on the matrix used. Results with PLA [217] and PE are very different [188]. Indeed, at low frequencies (low shear rates), the composite with PLA shows a plateau, whereas the composite with PE follows a power law for all frequencies. The addition of fibers appears to increase the material relaxation time and elasticity. This is shown in the study by Niang et al. using Typha fibers [218].

##### Water-Related Issues

Water resistance is an important property for composites. It can condition some specific uses of the material. Water can lead to the hydrolysis of materials, especially for polyesters (such as PLA or PET). The presence of water in polymers can have other consequences, such as swelling or the extraction of some molecules. However, although the hydrolysis resistance is not measured, water absorption is measured, using the standard NF EN ISO 62 [219]. It was shown that with a 30% loaded filament, layer thickness is proportional to water absorption (R² = 0.91) [177]. With a commercially fabricated PLA/wood filament, water absorption in 24 h was between 0.2 and 0.7%. These results are stable for up to 28 days. BambooFill filament water stability was measured [212], and the results show an almost 24% mass increase (800 h—33 days). This is explained by the hydrophilic nature of bamboo fibers (20%), but also by a bigger porosity of materials. In comparison, virgin PLA filaments and ABS filaments show lower water absorption rates. The use of thermomechanical pulp (paper mill production) with PLA also leads to an increase of water absorption from 1% to 6% [220]. Finally, tests on the hydrophobicity of the composites obtained show that sanding increases the contact angle, making the composite hydrophobic [221].

#### 4.5.4. Elastomers Thermoplastics

For a few specific applications (phone cases, for example), the incorporation of wood fibers in a thermoplastic elastomer like thermoplastic polyurethane (TPU) to produce a flexible material [222,223] is a promising solution. Unlike other polymers, TPU is a very flexible polymer. This is due to its low glass transition temperature (T_g_), around −16 °C [224]. So, the use of TPU is reserved for specific applications like flexible objects. Shape memory properties can be operated with TPU and wood fibers with a loading rate of 15% [89]. Mechanical properties are not comparable with rigid thermoplastics (higher elongation at break, for example).

#### 4.5.5. Challenges and Future Developments

To the best of the authors’ knowledge, there are no published data on composites with more than 60% wood. The highest loading rates were referenced at 60 wt.% for PLA [210] and TPU [222] and at 45 wt.% for HDPE [225]. With lower loading rates, more research can be found with 30% [87,213] and 20% or less [226]. Different patents are available, proving that using wood in material extrusion can be industrialized [227,228]. Commercial filament spools with maximum fill rates are around 40% [229,230].

Different species have been tested for 3D printing, such as beech [175,211], birch [87], pine [225,226,231], etc. Wood derivatives can also be used, such as lignin [209] or wood pulp from the paper industry [232]. Finally, reusing wood waste from sawmills (wood particles) [226,233] and from medium-density fiberboard (wood fibers mixed with adhesives) [234] as a new resource has been tested.

One of the reasons that FFF technology is the most famous is the availability of the process on the market. 3D printers can be easily found on the market for different prices. Moreover, products such as filament or pellets are available too. The leader for FGF pellets is Pollen Additive Manufacturing, with a 15% load rate for Woodfill pellets [235]. Many filaments with wood or sawdust with moderate loadings rates (less than 20 wt% wood filler) for FFF 3D printers are available on the market) [236,237]. However, some filaments have greater loading rates [230,238,239].

## 5. Conclusions

Wood is a promising material for being used as additive in 3D printing composites. Wood composites obtained with 3D printing processes have several uses. Several conclusions can be drawn from the state of the art described above.

**Figure 10:**
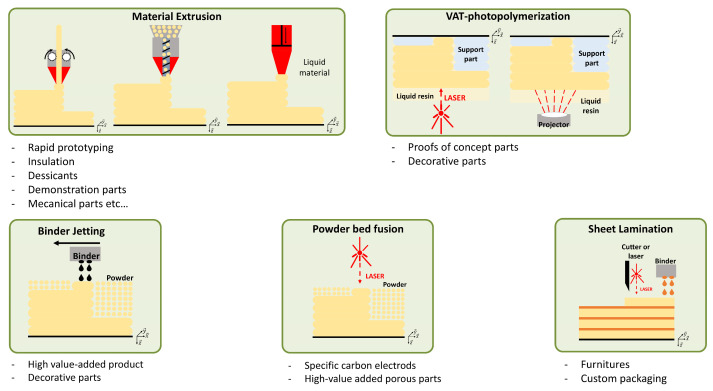
Prospective applications areas for the different 3D printing processes using wood.

- Wood species, compartment, and particle size affect the properties of the printing composite.
- Wood-based composites for 3D printing can be used in several markets where they are not currently present. PLA is the most widely used polymer in combination with wood. To increase the biodegradability of the polymer, other polymers of biological origin, such as PHA, could replace PLA.
- 3D printing is mainly used with untreated wood, and only a few studies on the use of wood waste are available. There is clearly a need to step up research into the decontamination of wood waste, to produce a raw material that is non-hazardous and eco-responsible.
- The most mature process at present is material extrusion (most specifically FFF). The challenges for this technology are to increase the loading rates and properties of the obtained parts and to open new potential markets.
- For improving the speed of printing and allowing transfer at an industrial scale, FGF is an interesting alternative to FFF. However, many technical issues remain to be fixed to allow this transfer.
- Photopolymerization, although producing very attractive renderings, still needs to be studied further, to enable higher filler contents to be obtained, while at the same time working on obtaining bio-sourced resins at an industrial level.
- For many applications, mechanical, rheological, and other properties are expected. The incorporation of compatibilizers or pre- and post-treatments of the parts obtained have still to be studied and improved.
- Each process has potential applications for future development as shown in Figure 10.

While wood is still used in the formulation of composites, the use of wood derivatives obtained by deep extraction (lignin, cellulose) can also increase the development of 3D printing and the recycling of wood waste. 3D printing using wood or its derivatives as a raw material is therefore a virtuous method for contributing to the major environmental issues at stake. Nevertheless, further research is needed to improve product life cycles, through higher loading rates and more environmentally friendly matrices. Finally, the challenges of recycling and end-of-life materials must be considered to fully embrace eco-design approaches.

## Figures and Tables

**Table 1:** National schemes for wood waste classification. For each classification, the relevant country codes are indicated above the definition [6,8].

Class A	Class B	Class C	Class D
**AT, BA, FR, NL, UK**	**AT, FR, FI, GE, NL, UK**	**AT, NL**	**FI, FR, GE, NL, SI SW, UK**
Unpainted and untreated wood, without or minor defects and a few restrictions on use	Partially contaminated (painted, varnished, coated, glued etc.) wood without preservatives or halogenated compounds	Hazardous waste wood—containing hazardous or toxic substances (e.g., paint, varnish, stain) or treated with halogenated organic materials	Wood waste treated with wood preservatives containing hazardous substances (e.g., copper and chromium or copper, chromium, arsenic, and creosote)
**FI, GE, SW**	**BA, CH**	**BA, FI, FR, GE, SW, UK**	**AT, BA**
Pure wood, only mechanically treated, insignificantly contaminated with harmful substances	Wood of average equivalent to A class quality, without specific requirements for pure wood	Non-hazardous waste wood with low-concentration chemical additives and organic halogenated compounds in the coating, with no wood preservatives	Wood waste that could not be assigned to other categories or components that are not wood waste (e.g., furniture with less than 50 wt.% wood)

AT: Austria; BA: Bosnia and Herzegovina; FR: France; NL: Netherlands; United Kingdom; FI: Finland; GE: Georgia; SI: Slovenia; SW: Sweden.

**Table 2:** Examples of composites among the many polymer/reinforcement combinations used in 3D printing.

3D Printing Process	Matrix Used (Supplier)	Reinforcement (Grain Size)	Key Results	Reference
Binder Jetting	Binder from ExOne GmbH (PM-B-SR2-02)	Frass from European house borer and termites (600–1000 µm)	Feedstock suitable for 3D printing	[84]
	HP binder	Miscanthus, wood, seashell, fruit stone, and rice husk flour	Large variety of renewable reinforcement available	[85]
Material Extrusion	PLA—Ingeo 4032D (NatureWorks)	Aspen wood flour (14 µm)	Thermal degradation is about 270 °C	[86]
	HDPE (Ra-Plast)	Yellow birch (500 µm)	HDPE with wood reduces 3D printing issues	[87]
	PHA	Wood flour	There are connections between process parameters and quality	[88]
	Elastollan C85A	Poplar wood flour (150 µm)	Flexible parts with wood flour can be 3D printed with shape memory properties	[89]
	ABS (Martogg Group)	Australian hardwood (90–212 µm)	A composite with 29 wt.% wood can be 3D printed with material extrusion	[90]
Powder Bed Fusion	Copolyester hot-melt adhesive	Pine powder (45–90 µm)	Loading rates like to material extrusion process can be obtained	[91]
	Poyether sulfone (PES)	Pine powder (45–90 µm)	Adding carbon nanotube can increase mechanical properties	[92]
	Phenolic Resin	Pine powder	Custom electrodes with wood can be successfully printed with wood	[93]
Vat Photopolymerization	RS-F2GPCL-04 (Formlabs)	Softwood kraft lignin	Lignin reduces cross-link reaction leading to more residual resin	[94]

## Data Availability

As this manuscript is a review, all data are included in this draft.
